# Relationship between Quality and Editorial Leadership of Biomedical Research Journals: A Comparative Study of Italian and UK Journals

**DOI:** 10.1371/journal.pone.0002512

**Published:** 2008-07-02

**Authors:** Valerie Matarese

**Affiliations:** UpTo Infotechnologies, Vidor (TV), Italy; Johns Hopkins Bloomberg School of Public Health, United States of America

## Abstract

**Background:**

The quality of biomedical reporting is guided by statements of several organizations. Although not all journals adhere to these guidelines, those that do demonstrate “editorial leadership” in their author community. To investigate a possible relationship between editorial leadership and journal quality, research journals from two European countries, one Anglophone and one non-Anglophone, were studied and compared. Quality was measured on a panel of bibliometric parameters while editorial leadership was evaluated from journals' instructions to authors.

**Methodology/Principal Findings:**

The study considered all 76 Italian journals indexed in Medline and 76 randomly chosen UK journals; only journals both edited and published in these countries were studied. Compared to UK journals, Italian journals published fewer papers (median, 60 vs. 93; *p* = 0.006), less often had online archives (43 vs. 74; *p*<0.001) and had lower median values of impact factor (1.2 vs. 2.7, *p*<0.001) and SCImago journal rank (0.09 vs. 0.25, *p*<0.001). Regarding editorial leadership, Italian journals less frequently required manuscripts to specify competing interests (*p*<0.001), authors' contributions (*p* = 0.005), funding (*p*<0.001), informed consent (*p*<0.001), ethics committee review (*p*<0.001). No Italian journal adhered to COPE or the CONSORT and QUOROM statements nor required clinical trial registration, while these characteristics were observed in 15%–43% of UK journals (*p*<0.001). At multiple regression, editorial leadership predicted 37.1%–49.9% of the variance in journal quality defined by citation statistics (*p*<0.0001); confounding variables inherent to a cross-cultural comparison had a relatively small contribution, explaining an additional 6.2%–13.8% of the variance.

**Conclusions/Significance:**

Journals from Italy scored worse for quality and editorial leadership than did their UK counterparts. Editorial leadership predicted quality for the entire set of journals. Greater appreciation of international initiatives to improve biomedical reporting may help low-quality journals achieve higher status.

## Introduction

Quality reporting of biomedical research is guided by statements from several organizations, most notably the International Committee of Medical Journal Editors (ICMJE, www.icmje.org). The ICMJE's “Uniform requirements for manuscripts submitted to biomedical journals” (URM) [1] provides guidance on manuscript preparation and on ethical issues related to publishing, for example authorship, conflict of interest, and internationally accepted ethical principles for research on humans and animals. These and other aspects of good research reporting are also dealt with by the Council of Science Editors (CSE, www.councilscienceeditors.org) [2], the European Association of Science Editors (EASE, www.ease.org.uk), the World Association of Medical Editors (WAME, www.wame.org) and the Committee on Publication Ethics (COPE, www.publicationethics.org.uk) [3]. Moreover, guidelines have been developed to improve the reporting of specific types of studies, such as the CONSORT statement (www.consort-statement.org) for randomized controlled trials (RCTs) [4] and the QUOROM statement for meta-analyses of RCTs [5]. Altogether, these recommendations assist both authors and journal editors in producing research papers that conform to current best practices; this is believed to promote good research, increase transparency [6], [7] and make the literature easier to assess [4], [8].

A journal that adheres to these recommendations may indicate so in its instructions to authors. In fact, both URM [1] and CONSORT [4] encourage adhering journals to indicate so in the instructions to authors, and COPE [3] suggests that journals use the instructions to inform authors about specific editorial policies. The instructions to authors and related editorial policy statements are documents in which journals typically present themselves and provide information about formatting a manuscript [9]. These are also documents to which authors can refer for guidance on the scientific content of papers. However, the amount of guidance provided varies greatly [9]. Well formulated instructions distinguish a journal for professionalism and rigor and may be considered as evidence of “editorial leadership”. Whether a high level of editorial leadership results in a better quality journal is unknown.

To investigate a possible relationship between journal quality and editorial leadership–as demonstrated in the instructions to authors, biomedical research journals from the UK and Italy were assessed and compared. These two European nations are similar in population and gross domestic product [10]. Both are members of the Group of Eight Industrialized Nations and are considered “scientifically advanced” [11]. Both also have legislation regarding ethical practices in biomedical research, as established by European directives [12]. However, the countries differ in language, culture and other characteristics relevant to research and publishing. For example, many initiatives to promote quality reporting, such as the EQUATOR network [8] and COPE, have roots in the UK. The UK spends almost twice as much as Italy on research and development [13], [14]. In Italy, underfunded research and low use of meritocracy [15] lead to “brain drain” (i.e. emigration of the best researchers to countries with better research environments), a phenomenon characteristic of Italian science [16]. Italy has no leading general medicine journal, like the *BMJ* and *Lancet* in the UK. Finally, unlike the UK, Italy has no member journal in the ICMJE and no affiliate in the World Medical Association, which produces the Declaration of Helsinki. These differences could negatively influence journal production in Italy and might be reflected in a lack of editorial leadership.

Therefore, journals from Italy and the UK were together considered to represent a wide range of quality within a European framework and were selected for this study on editorial leadership. One aim of the study was to test the hypothesis that journal quality is associated with editorial leadership. A second aim was to survey Italian journal production and, using UK journals as a reference, to identify areas in which journals from Italy, and possibly other non-Anglophone countries with similar scientific and publishing profiles, could improve.

## Materials and Methods

This study focused on research journals indexed in Medline. Italian journals were defined as journals whose editor-in-chief and publisher (or publishing office for multinational companies) were both in Italy. UK journals were similarly defined as those whose editor and publisher were both in the UK. In journals with co-editors, the presence of one editor in the country of interest was accepted.

### Journal selection

Candidates were identified from the “List of journals indexed in Medline, January 2006” of the US National Library of Medicine (NLM). This list categorizes journals only according to the publisher's country, and is not necessarily current (unpublished observation). Journals were excluded if only selected content was indexed, or if they were abstracting journals, supplements or review journals (reviews >50% of total papers). Additional candidate journals for Italy were identified from web research and personal knowledge. Candidates were included if information in their websites confirmed their eligibility regarding both editor and publisher; journals were not contacted to confirm the information presented online. If a journal did not have a web presence, its instructions to authors and editorial information were sought from other online sources (e.g. Mulford Library's database of instructions to authors, http://mulford.meduohio.edu/instr/index.html) and by contacting the editor-in-chief. Journals that had ceased publication after January 2006 were included only if sufficient information was still available online.

All Italian journals meeting these criteria were selected for study. Since there are 10 times more UK than Italian journals in Medline, an equivalent number of UK journals was selected. This was achieved by ordering the list of journals from England with computer-generated random numbers and screening the journals against inclusion and exclusion criteria until the required number was identified. The 33 Medline-listed journals from Scotland and Northern Ireland were not included in this selection, but journals with editors anywhere in the UK were included in the study.

The study was conducted between December 2006 and October 2007. Data were archived in a database programmed for this purpose using MySQL, and were viewed in a web-based interface written in PHP and HTML.

### Journal characterization

Since no single index of quality is widely accepted, journals were scored on a panel of bibliometric features related to quality (Table 1). These included, in addition to the impact factor (IF) and other citation statistics in the SCImago database (www.scimagojr.com) [17], the internationality of the editorial board and of the authorship, the types of papers published, and the availability of online archives and other features that enhance a journal's value to readers and authors.

**Table 1:** Bibliometric parameters used to characterize and assess the quality of Italian and UK journals.

Parameter	Description	Source
Publishes research on humans	At least one paper in 2000–2007 indexed with the MeSH term “human”	PubMed
Publishes research on animals	At least one paper in 2000–2007 indexed with the MeSH term “animal” excluding the subcategory “human”	PubMed
Size	Mean number of papers per year, 2000–2007	ESearch^a^
Start year	First year of publication	NLM Journals Database
International editorial board	At least one member from a country different from the publishing country	Journals' websites
International authorship	Percentage of articles in 2005 (excluding letters) with first author from a country different from the publishing country	PubMed
Language	A journal was considered to publish in a particular language if at least 10% of articles in 2005 were in that language	PubMed
Online archive	Electronic collection of all published articles from any point in time to the present; back files not continuous with the present excluded	Journals' online archives
PubMed link	Direct link from articles in PubMed to the journal's online archive	PubMed
Archive coverage	Number of years consecutive with the present in which archive coverage is complete	Journals' online archives
Archive access		
Open access	Published papers freely available immediately upon publication	Journals' online archives
Open access after embargo	Published papers freely available 6–24 months after publication	Journals' online archives
Publication type		
Letters	Expressed as percentage of all papers published in 2000–2007 (all journals)	ESearch^a^
Randomized controlled trials	Expressed as percentage of all clinical trials published in 2000–2007 (for journals that published at least one clinical trial)	ESearch^a^
Impact factor	2006	Journal Citation Reports^c^
SCImago journal rank	2006	SCImago^b^
H index	1996–2006	SCImago^b^
Cites/document	2006	SCImago^b^

a ESearch function of Entrez programming E-Utilities, eutils.ncbi.nlm.nih.gov.

b www.scimagojr.com.

c Thomson Scientific.

Editorial leadership was assessed on the basis of the most recent instructions to authors and editorial policy statements. Thirteen parameters were scored as yes or no:

- Adheres to ICMJE's “Uniform requirements for manuscripts submitted to biomedical journals”
- Defines authorship as “substantial contribution” or “scientific responsibility”
- Inquires about individual authors' contributions
- Requires manuscripts to indicate:
-
  - Sources of funding or sponsorship
  - Disclosure of conflict of interest or competing interest
  - Research adhered to the Declaration of Helsinki
  - Institutional ethics committee approved the study
  - Informed consent obtained from study participants
  - Clinical trial registration number
  - Study adhered to animal research laws
- Adheres to CONSORT statement
- Adheres to QUOROM statement
- Adheres to guidelines of the Committee on Publication Ethics

Finally, to understand differences in editorial leadership among journals, the membership lists of WAME (2006; from Internet Archive, www.archive.org), EASE (kindly provided by the EASE Council) and CSE (available to members) were searched for persons who indicated a professional appointment with a journal in the study.

### Statistical analysis

Associations between country group and categorical parameters were tested for significance using Pearson's chi-square test or Fisher's exact test. Differences between groups in continuous parameters were tested using the Mann-Whitney U test. Multiple regression analysis was performed using citation statistics as separate dependent (criteria) variables and 12 points of editorial leadership (excluding adherence to animal research laws) as predictor variables. To test the effect of confounders inherent to a cross-cultural comparison, additional predictor variables added to the model were country of origin, publishing language, size, presence of an international editorial board, and extent of international authorship (defined in Table 1). This analysis was performed only for journals that published research on humans in the period 2000–2007; in this way, purely clinical journals were not penalized for omitting information about animal research ethics, and the analysis was not confounded by non-clinical journals that ignored issues of clinical research ethics.

Statistical analyses were done using InStat (version 3.0b for Macintosh, GraphPad Software, San Diego, USA). Significance for two-tailed tests was set at *p*<0.05.

## Results

The NLM's List of journals indexed in Medline named 92 journals from Italy and 926 from the UK. Of the journals from Italy, 20 were excluded: 8 had ceased publication and no editorial information was found, 1 was only selectively indexed, 4 were not research journals, and 7 lacked an Italian editor or publisher. Four additional Medline-indexed Italian journals were included: 1 was absent from the NLM list, 1 was incorrectly listed under Germany and another 2, published by Elsevier (Netherlands), were edited in Italy and represented at least one Italian medical association. Although these latter two journals are not published in Italy, they were selected to better represent journal editing in Italy. Thus, 76 Italian research journals were included in the study, as were 76 journals from the UK (Appendix S1). To identify these latter journals, 180 candidates were randomly screened and 104 (58%) were excluded, mostly for being edited outside the UK. This exclusion rate permits an estimate of 389 journals edited and published in the UK. Therefore, the 76 UK journals in this study comprise 20% of all Medline-indexed research journals edited and published in the UK.

The two groups were similar in terms of the numbers of journals publishing research involving both humans and animals (biomedical), involving only humans (clinical), or not involving humans (e.g. animals or plants) (Table 2). However, Italian journals published fewer articles annually (median, 60 vs. 93; *p*<0.006) and were older (*p*<0.001) than UK journals. In particular, 8 Italian journals had been started since 2000 compared to 20 UK journals. These results suggest different trends in innovation and turnover. Almost all UK journals had an international editorial board and, although 47 Italian journals also did, this difference was significant (*p*<0.001). Most articles published in UK journals had a non-national first author, whereas only 37% of examined articles in Italian journals were authored internationally (*p*<0.001). These results suggest that UK journals can be considered international, while Italian journals have a tendency to being international, as also evidenced by their preference to publish exclusively in English. Concerning use of the Internet, Italian journals less frequently had online archives or links from PubMed to the archives. In particular, 17 of 43 Italian journals with online archives lacked PubMed links, vs. 6 of 74 UK journals. Despite being older, Italian journals offered archives covering fewer years (median, 6 vs. 10; *p*<0.001). However, Italian journals were more likely to offer open access and less likely to impose an embargo period, a phenomenon almost exclusive to UK journals. Regarding type of articles, journals from the two countries published similar, low percentages of letters to the editor (*p* = 0.070), but in Italian journals clinical trials were less frequently randomized and controlled (*p* = 0.029). Regarding citation, fewer Italian journals were indexed for IF (28 vs. 54, *p*<0.001) and those indexed had a lower median IF (1.2 vs. 2.7, *p*<0.001). All UK journals and 75 Italian journals were indexed in the SCImago database. Italian journals scored significantly lower in SCImago journal rank, H index and number of citations per document (*p*<0.001). Together, these results show that Italian journals are “smaller” and score lower for quality than UK journals.

**Table 2:** Characteristics of all Italian (IT) research journals and a randomly selected group of UK research journals indexed in Medline.

Characteristic	IT journals (n = 76)	UK journals (n = 76)	*p*
Publishes research involving, n			0.415†
Both humans and animals	53	49	
Humans only	20	20	
Animals only	3	1	
Neither	0	6	
No. of articles/year, 2000–2007, median (IQR)	60 (37–92)	93 (45–193)	0.006*
Start year, median (IQR)	1983 (1953–1993)	1992 (1983–2000)	<0.001*
International editorial board, n	47	70	<0.001†
% of articles authored internationally, 2005, median (IQR)	37 (7–62)	83 (62–91)	<0.001*
Language, n			<0.001†^a^
English	45	76	
English+Italian	22	0	
Italian	9	0	
Online archive, n	43	74	<0.001†
PubMed link to online archive, n	26	68	<0.001†
Archive coverage in years, median (IQR)	6 (4–7)	10 (7–16)	<0.001*
Archive access, n			<0.001†
Open access	18	13	
Embargo (≤24 months)	1	20	
Payment required	24	41	
No online archive	33	2	
Article types, 2000–2007, median (IQR)			
Letters, % of all articles	1.5 (0.5–3.6)	0.6 (0–3.8)	0.070*
RCTs, % of clinical trials^b^	36.1 (20.8–49.4)	44.7 (25.9–65.7)	0.029*
Indexed for impact factor, 2006, n	28	54	<0.001†
Impact factor, 2006, median (IQR)	1.2 (0.9–1.7)	2.7 (1.5–3.7)	<0.001*
Indexed in SCImago database, n	75	76	1.000§
SCImago journal rank, median (IQR)	0.09 (0.06–0.15)	0.25 (0.11–0.52)	<0.001*
H index, median (IQR)	10.0 (8.0–18.0)	23.5 (13.8–45.5)	<0.001*
Cites/document (2 years), median (IQR)	0.86 (0.45–1.39)	2.32 (1.25–3.57)	<0.001*

^a^ For the comparison English-only vs. not English-only; ^b^ For 72 IT and 59 UK journals that published at least one clinical trial in 2000–2007. ^*^ Mann-Whitney U test; ^†^ chi-square test; ^§^ Fisher's exact test. *IQR*, interquartile range; *RCT*, randomized controlled trial.

Each journal's instructions to authors and editorial policy statements were examined to assess the editorial leadership demonstrated towards authors (Table 3). Italian journals were significantly more likely than UK journals to declare to adhere to URM (27 vs. 11, *p* = 0.003). However, 24 Italian journals cited an outdated version (1997 or earlier) and 3 provided no reference; only 3 linked to the ICMJE website. UK journals also failed to cite the current version (5, no citation; 6, outdated versions) but 7 provided links. Fewer Italian journals based authorship on a “substantial contribution” or “scientific responsibility” (*p* = 0.019) or required that manuscripts specify authors' contributions (*p* = 0.005), funding or sponsorship (*p*<0.001), and competing or conflicting interests (*p*<0.001). For journals publishing human research, similar low numbers required that manuscripts state that research adhered to the Declaration of Helsinki: 9 Italian journals referred to outdated versions (1983 or earlier) and only 1 Italian and 6 UK journals referred or linked to the current version. Fewer Italian journals inquired about ethics committee review (*p*<0.001). Some inquiry about informed consent was made by 16 Italian and 42 UK journals (*p*<0.001). Of these, 7 Italian and 20 UK journals required informed consent for the publication of personal data, and 7 and 9 journals, respectively, required informed consent for patients' participation in clinical trials. No Italian journal required registration of clinical trials vs. 20 UK journals (*p*<0.001). For journals publishing animal research, fewer Italian journals inquired about adherence to animal research laws (*p* = 0.082). No Italian journal adopts CONSORT or QUOROM, while 15 UK journals follow CONSORT and 11 QUOROM. No Italian journal but 33 UK journals adhere to the guidelines of COPE. These results document that Italian journals show less editorial leadership than their UK counterparts, but that UK journals have room for improvement.

**Table 3:** Editorial leadership demonstrated by Italian (IT) and UK journals indexed in Medline, as apparent from instructions to authors and other editorial policy statements. Values are numbers of journals.

Characteristic	IT journals (n = 76)	UK journals (n = 76)	*p* ^a^
Adopts ICMJE uniform requirements	27	11	0.003
Defines authorship as “substantial contribution” or “scientific responsibility”	22	36	0.019
Inquires about individual authors' contributions	1	11	0.005
Requires statements about			
Funding or sponsorship	29	55	<0.001
Conflict of interest	22	48	<0.001
Adherence to Declaration of Helsinki	35	35	0.740^b^
Ethics committee review	19	50	<0.001
Informed consent	16	42	<0.001^b^
Registration of clinical trials	0	21	<0.001^b^
Adherence to animal research laws	26	33	0.082^c^
Adheres to CONSORT statement	0	15	<0.001^b^
Adheres to QUOROM statement	0	11	<0.001^b^
Adheres to COPE guidelines	0	33	<0.001

^a^ Chi-square test; ^b^ For journals that publish research on humans; ^c^ For journals that publish research on animals.

To further examine the relationship between editorial leadership and quality and to understand the impact of potential confounders inherent to a cross-cultural comparison, multiple regression analysis was performed for the 73 Italian and 69 UK journals that published research on humans (Table 4). When SCImago journal rank was used to define quality, the 12 parameters of editorial leadership considered as predictors explained about 37% of the variance in quality among the journals (multiple R = 0.609, *p*<0.0001). Each potential confounding variable added singly to the model had a minimal effect, and when all 5 confounders were added the ΔR^2^ was only 6.2%. With SCImago “cites/doc” as the definition of journal quality, editorial leadership explained almost 50% of the variance; again, the possible confounding factors had a small effect and together increased R^2^ by 13.8%. Finally, for the subset of journals indexed for IF, editorial leadership explained 49.9% of the variance in this parameter and confounders increased this to 57.4%. These results suggest that editorial leadership is intimately associated with journal quality as assessed by various citation statistics, and that the impact of variables such as country of origin, publishing language and internationality is not strong.

**Table 4:** Multiple regression analysis of the impact of editorial leadership and of potential confounding parameters on journal quality, for 73 Italian and 69 UK journals that publish research on humans.

Predictors	SCImago journal rank		SCImago cites/doc		Impact factor	
	R^b^	R^2c^	R^b^	R^2c^	R^b^	R^2c^
Editorial leadership^a^	0.609	37.1*	0.707	49.9†	0.707	49.9‡
+Publishing language	0.613	0.4	0.713	0.9	–^d^	–^d^
+International editorial board	0.616	0.8	0.738	4.5	0.721	2.0
+Country of origin	0.620	1.3	0.721	2.0	0.718	1.6
+Size (articles/year)	0.629	2.4	0.743	5.4	0.733	3.8
+% articles with international authorship	0.636	3.3	0.734	4.0	0.726	2.8
+All potential confounders	0.658	6.2	0.798	13.8	0.757	7.5
Total explained variance, %		43.3		63.7		57.4

Independent analyses were run for the criteria variables SCImago journal rank and SCImago cites/doc (available for 141 of the journals) and impact factor (75 journals).

^a^ All aspects but adherence to animal research laws were considered. ^b^ Multiple correlation coefficient for all predictors included in the model; coefficients for all models were statistically significant (*p*<0.0001). ^c^ Percentage of variance explained by inclusion of the new predictor in the model. ^d^ Language not entered into the model because the IF journal set has only one bilingual journal and no journals in Italian.

* F_(141,128)_ = 6.28; ^†^F_(141,128)_ = 10.6; ^‡^F_(75,62)_ = 5.15.

Finally, regarding the participation of journals in associations for editors, one Italian and 20 UK journals are affiliated with WAME. Similarly, one Italian and 12 UK journals have editors or staff who are members of EASE. Only one UK journal has staff listed as member of CSE. Thus, Italian journals have few means of learning about the latest international standards and trends in biomedical publishing, but many UK journals are also missing this opportunity. The lower participation of Italian editors may have contributed to the lower performance of Italian journals on parameters of editorial leadership examined in this study.

## Discussion

In this study, Medline-indexed Italian research journals scored lower for quality than did their UK counterparts. Italian journals also scored worse on individual parameters of editorial leadership, even though UK journals have room for improvement. Additionally, when Italian and UK journals were assessed together by multiple regression analysis, parameters of editorial leadership explained 37.1%–49.9% of the variation in citation statistics, taken as indicators of quality. Although these data cannot be taken to imply a causal relationship, they suggest that–for lower-ranked journals wishing to attain higher status–an appropriate goal would be to provide greater editorial guidance to the author communities they serve.

A unique feature of this study is its focus on research journals produced in two countries. Previous studies on editorial practices and quality focused instead on leading journals (i.e. selected subjectively or by IF [9], [18]) or on randomly sampled journals [19], but lacked comparison groups. The comparison between two European countries provides insight into the editorial challenges faced by journals from non-Anglophone industrialized countries. Furthermore, since the selection criterion in this study was indexing in Medline, rather than IF, the samples are larger and better representative of the quality and diversity of biomedical research journals from the two countries. Finally, only journals both edited and published in the countries of interest were considered. This dual requirement excluded 58% of Medline-indexed journals listed as being from the UK and 8% of journals listed as being from Italy. Thus, when characterizing journals (and national research output), especially from the UK, it is important to verify country data and not rely on information provided by sources that only use the publisher's country of origin.

While the findings of lower quality and editorial leadership among Italian journals may have been expected [20], [21], the outcome for UK journals was surprising. In particular, few UK journals (14%) declared to adhere to URM. This rate is significantly lower than that for Italian journals (36%) and is also lower than the 41% reported by Schriger et al. [9] in a survey of instructions to authors from 166 leading general and specialty clinical journals. Moreover, just less than half of UK journals (like Italian journals) requested manuscripts to indicate that research adhered to the Declaration of Helsinki, despite the fact that most journals in both groups publish research on humans. Notwithstanding these low rates, UK journals often adhere to specific items in both URM and the Declaration of Helsinki, such as requiring manuscripts to indicate sources of funding (72%), demonstrate approval by ethics committees (66%), disclose competing interests (63%), and document informed consent (55%). The finding that 20% of randomly sampled UK journals endorses the CONSORT statement agrees with the 22% observed by Schriger et al. [9]. That 63% of UK journals had a written policy on the disclosure of conflict of interest is a positive finding, higher than the 33% found for 84 high-IF journals from 12 scientific disciplines [18]. Why few UK journals–and even fewer Italian journals–provide authors with adequate guidance on the scientific content of papers is unknown: they may trust authors and peer reviewers to handle the issues, they may ignore the issues entirely, or they may believe that setting high standards will reduce the number of submissions [9]. This was the case for one editor from a developing country, who feared that adhering to the requirement for clinical trial registration would result in authors sending their papers to journals with lower standards [22].

This study is limited by its observational design, which is unable to demonstrate a causal relationship between editorial leadership and journal quality. Another limitation is that the study only evaluated leadership as expressed through the instructions for authors and did not investigate actual editorial practices, such as systems of in-house review, peer review and technical copyediting; thus, it may have underestimated leadership in journals with “author-helpful” policies [23]. Additionally, this research used publicly available information, rather than interviews or questionnaires. Judging a journal by its written policies may not completely describe its editorial practices, as highlighted by the study of 84 high-IF science journals, of which 11 had only unpublished conflict of interest policies [18]. Furthermore, journals do not always adhere to their written policies, as shown by a survey of 5 leading general medicine journals: despite adequate instructions, 31% of published papers did not mention ethics committee approval and 47% did not mention informed consent [24]. Finally, only 20% of the total estimated number of UK journals was evaluated; thus this study provides a full survey of Italian journal production only.

Nation-wide surveys of journal production are rarely performed. Instead, the scientific output of nations is evaluated from the numbers of published papers and their citations [13], [25] or from the journals in which papers are published (e.g. [26], [27]). These evaluations emphasize publishing in high-IF journals, nearly all of which are based in Anglophone countries. Although such journals are considered “international”, they have limited coverage of health issues relevant to the developing world [28]. This criticism has encouraged a new appreciation for the real and potential contribution of “local” journals–especially in resource-poor countries–and has led to the creation of associations for editors in Africa and the Eastern Mediterranean [29]. In this panorama, journals from non-Anglophone industrialized countries (especially in Europe) have an unclear status. In a recent debate, journal production in Germany was given the status of a developing country, especially for difficulties in scientific communication [30]. Yet, as shown here, these journals can have a substantial international character and aim to an international audience. They may act as a bridge between mainstream science and the scientific periphery [31], by publishing papers from less rich countries, especially through editorial collaborations with Eastern Mediterranean and Asian medical associations [32], [33]. Although this author community may produce lower quality research (due to poor resources or research skills), the information can be internationally important [34]. Thus, it is imperative that these journals, like those from semi-developed nations [35], adopt good editorial practices, to improve their scientific quality as well as their effectiveness as an international voice for non-Anglophone authors. Assistance in reaching these goals is now available from the EQUATOR network [8].

In summary, this survey of journal production from two European countries demonstrated an association between journal quality and editorial leadership, defined as the guidance in a journal's instructions to authors. Journals from Italy performed worse than those from the UK, although these latter journals nonetheless have room for improvement. Insufficient editorial leadership implies lower expectations regarding manuscript preparation, and may generate a vicious cycle [34] in which authors of quality research are not attracted to submit manuscripts, obliging the journal to accept poorer quality papers. Whether journals–Italian ones in particular–can improve quality by improving instructions to authors is unknown. More likely, quality improvement requires editors to have greater appreciation of international initiatives promoting quality publishing and to act as educators in the scientific communities performing the research they publish. Improved instructions to authors may be the expression of the editor's understanding of this role.

## Supporting Information

Appendix S1 (0.12 MB DOC)
