# Supplementary material for: Relationship between Quality and Editorial Leadership of Biomedical Research Journals: A Comparative Study of Italian and UK Journals
Source: PLoS One. 2008 Jul 2;3(7):e2512. doi: 10.1371/journal.pone.0002512 (PMC2438474; doi:10.1371/journal.pone.0002512)
Supplement: Appendix S1 — (0.12 MB DOC) [file pone.0002512.s001.doc]

**Appendix S1** Medline abbreviated names of 76 Italian and 76 UK journals

### **Italian journals**

Acta Bio-medica

Acta Diabetol

Acta Myol

Acta Otorhinolaryngol Ital

Aging Clin Exp Res

Ann Chim

Ann Ig

Ann Ist Super Sanita

Ann Ital Chir

Ann Ital Med Int

Arch Ital Biol

Arch Ital Urol Androl

Assist Inferm Ric

Chir Ital

Clin Exp Med

Clin Exp Rheumatol

Clin Ter

Dig Liver Dis

Eat Weight Disord

Epidemiol Prev

Epidemiol Psichiatr Soc

Eur J Histochem

Eur J Ophthalmol

Eur Rev Med Pharmacol Sci

Eura Medicophys

Funct Neurol

G Chir

G Ital Med Lav Ergon

G Ital Nefrol

Haematologica

Infez Med

Int J Artif Organs

Int J Biol Markers

Int J Immunopathol Pharmacol

Ital Heart J

Ital J Anat Embryol

Ital J Biochem

J Biol Regul Homeost Agents

J Chemother

J Endocrinol Invest

J Exp Clin Cancer Res

J Headache Pain

J Ment Health Policy Econ

J Nephrol

J Neurosurg Sci

J Sports Med Phys Fitness

JOP

Med Lav

Med Secoli

Minerva Anestesiol

Minerva Cardioangiol

Minerva Chir

Minerva Endocrinol

Minerva Gastroenterol Dietol

Minerva Ginecol

Minerva Med

Minerva Pediatr

Minerva Stomatol

Minerva Urol Nefrol

Monaldi Arch Chest Dis

Neurol Sci

New Microbiol

Panminerva Med

Parassitologia

Pathologica

Pediatr Med Chir

Pharmacol Res

Prof Inferm

Q J Nucl Med Mol Imaging

Radiol Med Torino

Recenti Prog Med

Reumatismo

Riv Biol

Sarcoidosis Vasc Diffuse Lung Dis

Tech Coloproctol

Tumori

### **UK journals**

Aging Ment Health

Aliment Pharmacol Ther

Anaesthesia

Ann Oncol

Arch Oral Biol

Arthritis Res Ther

Avian Pathol

Behav Pharmacol

Biochem J

Biomarkers

Biomed Chromatogr

Biopharm Drug Dispos

BMC Bioinformatics

BMC Dev Biol

BMC Evol Biol

BMC Genet

BMC Geriatr

BMC Pharmacol

BMC Physiol

BMC Plant Biol

BMC Psychiatry

Br J Clin Psychol

Br J Community Nurs

Br J Pharmacol

Cancer Imaging

Cell Biochem Funct

Clin Exp Allergy

Clin Med

Comput Methods Biomech Biomed Engin

DNA Seq

Emerg Med J

Eur J Anaesthesiol

Eur J Cancer

Europace

Exp Physiol

FEBS J

Genome Biol

Gut

Hematology

Heredity

HIV Med

Insect Mol Biol

Int J Food Sci Nutr

Int J Palliat Nurs

Int J Psychiatr Nurs Res

J Antimicrob Chemother

J Biol

J Health Psychol

J Intellect Disabil

J Interprof Care

J Med Genet

J Neuroendocrinol

J Neurol Neurosurg Psychiatry

J Psychosom Res

J Sleep Res

J Wound Care

Knee

Lancet Neurol

Med Confl Surviv

Med Leg J

Mol Membr Biol

Neuropathol Appl Neurobiol

New Phytol

Nucl Med Commun

Nucleic Acids Res

Nurs Ethics

Paediatr Perinat Epidemiol

Pediatr Allergy Immunol

Physiother Res Int

Phytother Res

QJM

RCM Midwives

Sex Transm Infect

Stat Methods Med Res

Thorax

Yeast
